# Effect of oral antiviral treatment on long-term outcomes of radiofrequency ablation therapy for hepatitis B virus-related hepatocellular carcinoma

**DOI:** 10.18632/oncotarget.10026

**Published:** 2016-06-14

**Authors:** Won Sohn, Tae Wook Kang, Sun-Kyu Choi, Sin-Ho Jung, Min Woo Lee, Hyo Keun Lim, Ju-Yeon Cho, Sang Goon Shim, Dong Hyun Sinn, Geum-Youn Gwak, Moon Seok Choi, Joon Hyeok Lee, Kwang Cheol Koh, Seung Woon Paik, Hyunchul Rhim, Yong-Han Paik

**Affiliations:** ^1^ Department of Medicine, Samsung Medical Center, Sungkyunkwan University School of Medicine, Seoul, Korea; ^2^ Department of Hepatology, Bundang Jesaeng Hospital, Sungnam, Korea; ^3^ Department of Radiology, Samsung Medical Center, Sungkyunkwan University School of Medicine, Seoul, Korea; ^4^ Department of Biostatistics and Clinical Epidemiology, Samsung Medical Center, Seoul, Korea; ^5^ Department of Health Science and Technology, Samsung Advanced Institute for Health Science and Technology, Sungkyunkwan University, Seoul, Korea; ^6^ Department of Medicine, Chosun University Hospital, Gwang-Ju, Korea; ^7^ Department of Medicine, Samsung Changwon Hospital, Sungkyunkwan University School of Medicine, Changwon, Korea

**Keywords:** hepatocellular carcinoma, radiofrequency ablation, chronic hepatitis B, antiviral treatment

## Abstract

**Objectives:**

This study aimed to investigate the effect of oral antiviral treatment on the prognosis of hepatitis B virus (HBV)-related hepatocellular carcinoma (HCC) after radiofrequency (RF) ablation.

**Methods:**

Between January 2003 and December 2010, 228 patients without a history of antiviral treatment were treated with RF ablation for a single HBV-related HCC. We divided the patients into two groups, patients who received (n=125) or did not receive antiviral treatment (n=103), based on whether oral antiviral treatment was administered after RF ablation. The median duration of antiviral treatment was 60.1 months. HCC recurrence and overall survival were compared in the two groups in the full cohort and the propensity score-matched cohort.

**Results:**

In the matched cohort, the probability of HCC recurrence at 5 years was 43.8% for the non-antiviral treatment group and 14.7% for the antiviral treatment group (*p*<0.001). The probability of overall survival at 5 years was 77.2% for the non-antiviral treatment group and 93.5% for the antiviral treatment group (*p*=0.002). Multivariable analysis showed that risk factors for HCC recurrence included large tumor size (hazard ratio (HR)=1.30, *p*=0.022), HBV DNA serum level (HR=1.11, *p*=0.005), and serum AFP level ≥20 ng/mL (HR=1.66, *p*=0.005). Overall survival was associated with larger tumor size (HR=1.86, *p*=0.001) and Child-Pugh Class B (HR=2.13, *p*=0.019). Oral antiviral treatment after RF ablation was significantly associated with a lower risk of tumor recurrence and death (HR=0.33, *p*<0.001, and HR=0.44, *p*=0.004).

**Conclusion:**

Use of oral antiviral treatment after curative RF ablation was associated with favorable outcomes in terms of tumor recurrence and overall survival in patients with HBV-related HCC.

## INTRODUCTION

Hepatocellular carcinoma (HCC) is one of the most common cancers [1]. The preferred means of treatment is to detect the tumor at an early stage and to treat with a curative therapy, such as surgical resection, liver transplantation and local ablation [2]. Radiofrequency (RF) ablation is a representative local ablation therapy for HCC treatment. It is widely used to treat HCC at an early stage when the case is not a candidate for surgical therapies. Currently, RF ablation is recommended as a curative therapeutic modality for patients with very-early stage HCC according to the recently published Barcelona Clinic Liver Cancer treatment strategy [3].

The therapeutic outcomes of RF ablation for HCC are known to be associated with technical issues (e.g. incomplete ablation), tumor characteristics (e.g. tumor size, number, and location), and underlying liver status (e.g. cirrhosis and hepatic iron accumulation) [4–7]. Recent studies, including that carried out by our group, show that hepatitis viral load is an independent risk factor for prognosis of HCC after surgical resection or RF ablation in patients with chronic hepatitis B (CHB) [8–11]. High viral load at resection is associated with tumor recurrence of hepatitis B virus (HBV)-related HCC after resection [8–10]. In addition, our group reported that a high level of HBV DNA at RF ablation is a risk factor for tumor recurrence in HBV-related HCC after RF ablation [11]. Therefore, the control of viral replication may be important for prognosis after the treatment of HBV-related HCC. Recent studies demonstrate that oral antiviral treatment using nucleos(t)ide (nucleoside/nucleotide) analog results in favorable outcomes in terms of tumor recurrence and overall survival of HBV-related HCC after surgical resection [12–14]. However, a few studies have been carried out to explore on the effect of antiviral treatment on the prognosis of HBV-related HCC after curative RF ablation [15, 16]. Our group reported that the absence of antiviral therapy during follow up was associated with poor survival in HCC patients who received RF ablation as first-line therapy [15]. Recently, Taiwanese investigators reported that nucleos(t)ide analog treatment is associated with a decreased risk of HCC recurrence using nationwide health insurance database [16]. However, previous studies did not provide detailed HBV-related data such as pretreatment HBV DNA levels or HBeAg status that are known as important factors affecting prognosis in HBV-related HCC. Therefore we aimed to investigate the precise role of nucleos(t)ide analog treatment on the long-term prognosis of HBV-related HCC after curative RF ablation.

We compared long-term prognosis including tumor recurrence and overall survival between an oral nucleos(t)ide treatment group and a non-treatment group in HBV-related HCC after curative RF ablation. In addition, risk factors for tumor recurrence and overall survival of HBV-related HCC after RF ablation were analyzed.

## RESULTS

### Baseline characteristics

Baseline characteristics of the two groups are described in Table 1. While 103 patients did not receive antiviral treatment after RF ablation, 125 patients received antiviral treatment after RF ablation (entecavir 0.5mg/d, n=68; lamivudine 100mg/d, n=45; clevudine 30mg/d, n=7; adefovir 10mg/d, n=4; tenofovir 300mg/d, n=1). Supplemental Table 1 shows the stratified characteristics according to antiviral agents. There was no adverse event in patients treated with entecavir, lamivudine, and tenofovir. Mild renal insufficiency occurred in one patient treated with adefovir that was recovered after reducing to half dose. A patient experienced myopathy during clevudine use and was fully recovered after changing to entecavir. The initiation of antiviral treatment was decided according to the KASL guideline in 117 patients of the 125 patients (94%). However, the antiviral agents were administered to 8 patients (6%) who did not meet the KASL guideline according to a physician's recommendation. The median follow-up duration of the patients was 64.8 (interquartile range: 46.8-90.0) and 68.4 (interquartile range: 53.3-89.7) months in the non-antiviral treatment group and the antiviral treatment group, respectively. There was no significant difference in follow-up duration between the two groups. The median duration of antiviral treatment was 60.1 (interquartile range, 46.1-77.9) months. The median interval from RF ablation to the beginning of antiviral treatment was 3.4 (interquartile range, 0-16.5) months. There was no significant difference in age, gender, tumor size, platelet count, PT-INR, liver cirrhosis, and Child-Pugh class status between two groups. However, the antiviral treatment group had significantly higher levels of serum AST, ALT, HBV DNA and AFP than the non-antiviral treatment group (*p*=0.002, *p*=0.003, *p*<0.001, and *p*=0.007). Also, the number of patients with HBeAg positivity was significantly higher in the antiviral treatment group than in the non-antiviral treatment group (49% versus 22%, *p*<0.001). Of the 228 patients, histologic diagnosis for HCC was done in 28 patients (12.3%). The differentiation type of HCC was Edmondson-Steiner grade I in 10 patients, grade II in 17 patients, and grade IV in 1 patient.

**Table 1:** Baseline characteristics of all patients (n=228)

	Non-antiviral treatment group (n=103)	Antiviral treatment group (n=125)	P-value
Age (years)	55.2 ± 8.9	55.0 ± 8.9	0.856
Gender			0.328
women	23 (22%)	35 (28%)	
men	80 (78%)	90 (72%)	
Tumor size (mm)	2.1 ± 0.6	2.2 ± 0.7	0.464
Platelet (x10^3^/mm^3^)	111.9 ± 48.5	110.2 ± 45.7	0.792
Prothrombin time (INR)	1.21 ± 0.14	1.23 ± 0.16	0.347
Albumin (g/dL)	3.6 ± 0.5	3.6 ± 0.6	0.557
Total bilirubin (mg/dL)	1.0 ± 0.6	1.0 ± 0.6	0.721
AST (U/L)	43.1 ± 21.9	54.2 ± 31.8	0.002
ALT (U/L)	36.9 ± 32.3	52.1 ± 42.5	0.003
Log_10_HBV DNA ( IU/mL)	2.8 ± 2.6	5.3 ± 2.0	<0.001
AFP (ng/mL)			0.007
<20	57 (55%)	47 (38%)	
≥20	46 (45%)	78 (62%)	
HBeAg (N, %)			<0.001
negative	80 (78%)	64 (51%)	
positive	23 (22%)	61 (49%)	
Liver cirrhosis (N, %)			
absence	17 (17%)	21 (17%)	0.953
presence	86 (83%)	104 (83%)	
Child-Pugh class (N, %)			0.661
A	86 (83%)	107 (86%)	
B	17 (17%)	18 (14%)	
Antiviral therapy (N, %)			
Lamivudine		68 (54%)	
Entecavir		45 (36%)	
Clevudine		7 (6%)	
Adefovir		4 (3%)	
Tenofovir		1 (1%)	

Data are presented as the mean ± standard deviation or number of patients (percentages in parentheses).

*Abbreviations: RF, radiofrequency; INR, international normalized ratio; AST, aspartate aminotransferase; ALT, alanine aminotransferase; HBV, hepatitis B virus; AFP, alpha-fetoprotein; HBeAg, hepatitis B envelope antigen.

### Tumor recurrence and overall survival according to the use of antiviral treatment

Among all 228 patients, tumor recurrence and death were observed in 152 (66.7%) and 54 (23.7%) patients, respectively. Table 2 shows the comparison of baseline characteristics between the two groups after propensity score matching. After matching, 104 patients were included in the antiviral treatment group and 62 patients were included in the non-antiviral treatment group. There was no significant difference in all clinical variables including serum AST, ALT, HBV DNA, AFP, and HBeAg status between the two groups. In addition, the mean standardized difference of each variable indicated very small differences between the groups.

**Table 2:** Baseline characteristics of matched patients (n=166)

	Non-antiviral treatment group (n=62)	Antiviral treatment group (n=104)	P-value	Standardized mean difference
Age (years)	55.6 ± 9.0	55.5 ± 9.2	0.939	0.012
Gender			0.721	0.063
women	20 (32%)	31 (30%)		
men	42 (68%)	73 (70%)		
Tumor size (cm)	2.3 ± 0.6	2.2 ± 0.7	0.715	0.065
Platelet (x10^3^/mm^3^)	112.3 ± 47.3	111.4 ± 45.0	0.903	0.019
Prothrombin time (INR)	1.22 ± 0.15	1.22 ± 0.16	0.949	−0.011
Albumin (g/dL)	3.6 ± 0.4	3.6 ± 0.6	0.874	0.025
Total bilirubin (mg/dL)	1.0 ± 0.7	1.0 ± 0.6	0.876	0.025
AST (U/L)	46.9 ± 25.1	47.7 ± 22.3	0.828	−0.039
ALT (U/L)	42.2 ± 39.2	42.4 ± 21.9	0.958	−0.009
Log_10_HBV DNA ( IU/mL)	4.2 ± 2.5	4.4 ± 2.2	0.504	−0.091
AFP (ng/mL)			0.930	0.013
<20	28 (45%)	48 (46%)		
≥20	34 (55%)	56 (54%)		
HBeAg (N, %)			0.910	−0.021
negative	41 (66%)	68 (65%)		
positive	21 (34%)	36 (35%)		
Liver cirrhosis (N, %)			0.737	0.048
absence	7 (11%)	14 (13%)		
presence	55 (89%)	90 (87%)		
Child-Pugh class (N, %)			0.971	0.006
A	52 (84%)	87 (84%)		
B	10 (16%)	17 (16%)		

*Abbreviations: RF, radiofrequency; INR, international normalized ratio; AST, aspartate aminotransferase; ALT, alanine aminotransferase; HBV, hepatitis B virus; AFP, alpha-fetoprotein; HBeAg, hepatitis B envelope antigen.

**Figure 1:**
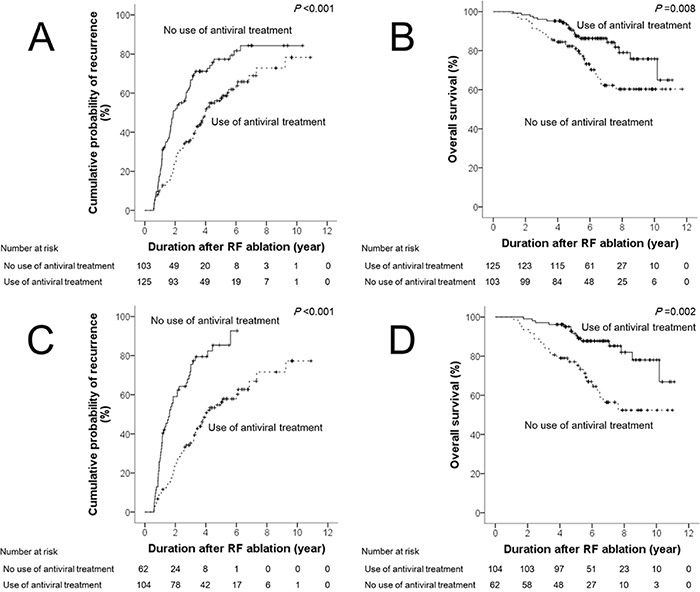
Differences in time to recurrence and overall survival according to the use of antiviral treatment after RF ablation in the full cohort **A & B.** and the matched cohort **C & D.** *Abbreviation: RF, radiofrequency.

We compared the differences in time to recurrence and overall survival according to the use of antiviral treatment after RF ablation in both the full and matched cohorts (Figure 1). In the full cohort, the probability of 1-year, 3-year, 5-year, and 10-year recurrence were 18.4%, 64.7%, 77.3%, and 100%, respectively, in the non-antiviral treatment group; and 10.4%, 35.9%, 56.1%, and 78.2%, respectively, in the antiviral treatment group (Figure 1A). The median time to recurrence was 1.9 (95% CI: 1.4-2.4) and 4.0 (95% CI: 3.3-4.7) years in the non-antiviral and antiviral treatment groups, respectively. Tumor recurrence was significantly different between the two groups (*p*<0.001). The probabilities of 1-year, 3-year, 5-year, and 10-year overall survival were 100%, 90.3%, 82.3%, and 60.3%, respectively, in the non-antiviral treatment group, and 100%, 96.8%, 90.5%, and 75.7%, respectively, in the antiviral treatment group (Figure 1B). The mean duration of overall survival was 8.9 (95% CI: 8.1-9.7) and 9.7 (95% CI: 9.1-10.3) years in the non-antiviral and antiviral treatment groups, respectively. Overall survival was significantly different between the two groups (*p*=0.008).

In the matched cohort, the probabilities of 1-year, 3-year, 5-year, and 10-year recurrence were 27.4%, 71.8%, 85.3%, and 100%, respectively, in the non-antiviral treatment group, and 8.7%, 32.8%, 56.2%, and 77.7%, respectively, in the antiviral treatment group (Figure 1C). The median time to recurrence was 1.6 (95% CI: 1.2-2.7) and 4.0 (95% CI: 3.6-6.8) years in the non-antiviral and antiviral treatment groups, respectively. Tumor recurrence was significantly different between the two groups (*p*<0.001). The probabilities of 1-year, 3-year, 5-year, and 10-year overall survival were 100%, 87.1%, 77.2%, and 52.4%, respectively, in the non-antiviral treatment group; and 100%, 97.5%, 93.5%, and 82.1%, respectively, in the antiviral treatment group (Figure 1D). The mean duration of overall survival was 6.1 (95% CI: 5.6-6.7) and 9.4 (95% CI: 8.9-9.9) years in the non-antiviral and antiviral treatment groups, respectively. Overall survival was significantly different between the two groups (*p*=0.002).

The recurrence events and therapeutic modalities until the end of the follow-up were demonstrated in Supplementary Figure 1 and Supplementary Table 2. Also, we checked the percentage of complete ablation in each event. The complete ablation rates were 95.9% (71/74) at the 1st event, 96.9% (31/32) at the 2nd event, and 100 % (9/9) at the 3rd event.

### Risk factors for tumor recurrence

Table 3 shows the risk factors for tumor recurrence of HBV-related HCC after RF ablation in the full cohort. Univariable analysis revealed that tumor recurrence was significantly associated with larger tumor size (hazard ratio (HR)=1.35 (95% CI: 1.08-1.68), *p*=0.008), increased PT-INR (HR=3.18 (95% CI: 1.27-7.95), *p*=0.013), a lower level of serum albumin (HR=0.65 (95% CI: 0.49-0.87), *p*=0.003), and serum AFP level ≥20 ng/mL (HR=1.53 (95% CI: 1.11-2.12), *p*=0.009). In addition, the use of antiviral treatment after RF ablation was associated with a significantly lower risk of tumor recurrence (HR=0.53 (95% CI: 0.38-0.73), *p*<0.001). Multivariable analysis revealed that tumor recurrence was associated with larger tumor size (HR=1.30 (95% CI: 1.04-1.63), *p*=0.022), higher serum HBV DNA level (HR=1.11 (95% CI: 1.03-1.20), *p*=0.005), and serum AFP level ≥20 ng/mL (HR=1.66 (95% CI: 1.17-2.36), *p*=0.005). Use of antiviral treatment after RF ablation was also significantly associated with lower risk of tumor recurrence (HR=0.33, (95% CI: 0.23-0.48), *p*<0.001).

**Table 3:** Univariable and multivariable analyses of risk factors for recurrence of HBV-related HCC patients after RF ablation in the full cohort (n=228)

	Univariable HR (95% CI)	*p*-value	Multivariable HR (95% CI)	*p*-value
Age (years)	1.00 (0.97-1.01)	0.547		
Men	0.98 (0.68-1.40)	0.908		
Tumor size (mm)	1.35 (1.08-1.68)	0.008	1.30 (1.04-1.63)	0.022
Platelet (x10^3^/mm^3^)	1.00 (0.99-1.00)	0.151		
Prothrombin time (INR)	3.18 (1.27-7.95)	0.013		
Albumin (g/dL)	0.65 (0.49-0.87)	0.003		
Total bilirubin (mg/dL)	1.21 (0.97-1.50)	0.092		
AST (U/L)	1.00 (0.99-1.01)	0.142		
ALT (U/L)	1.00 (0.99-1.01)	0.793		
Log_10_HBV DNA ( IU/mL)	1.05 (0.98-1.11)	0.171	1.11 (1.03-1.20)	0.005
AFP (≥20 ng/mL)	1.53 (1.11-2.12)	0.009	1.66 (1.17-2.36)	0.005
HBeAg (+)	1.32 (0.95-1.83)	0.094		
Liver cirrhosis (+)	1.34 (0.85-2.11)	0.205		
Child-Pugh class B	1.43 (0.94-2.18)	0.099		
Antiviral treatment after RF ablation (+)	0.53 (0.38-0.73)	<0.001	0.33 (0.23-0.48)	<0.001

*Abbreviations: HBV, hepatitis B virus; HCC, hepatocellular carcinoma; RF, radiofrequency; HR, hazard ratio; CI, confidence interval; INR, international normalized ratio; AST, aspartate aminotransferase; ALT, alanine aminotransferase; AFP, alpha-fetoprotein; HBeAg, hepatitis B envelope antigen.

In a subgroup analysis based on tumor size, multivariable analysis showed that use of antiviral treatment after RF ablation was associated with a significantly lower risk of tumor recurrence (HR=0.41 (95% CI: 0.24-0.69), *p*=0.001) in patients with tumor size <2 cm. In patients with tumor size ≥2 cm, multivariable analysis showed that tumor recurrence was associated with increased PT-INR (HR=8.72 (95% CI: 2.06-36.91), *p*=0.003), and serum AFP level ≥20 ng/mL (HR=1.83 (95% CI: 1.17-2.86), *p*=0.009). The use of antiviral treatment after RF ablation was associated with a significantly lower risk of tumor recurrence (HR=0.44 (95% CI: 0.28-0.70), *p*<0.001) (Table 4).

**Table 4:** Multivariable analysis of risk factors for tumor recurrence of HBV-related HCC after RF ablation in a subgroup of patients according to tumor size

	Tumor size <2 cm		Tumor size ≥2 cm	
	HR (95% CI)	*p*-value	HR (95% CI)	*p*-value
Prothrombin time (INR)			8.72 (2.06-36.91)	0.003
AFP (≥20 ng/mL)			1.83 (1.17-2.86)	0.009
Antiviral treatment after RF ablation (+)	0.41 (0.24-0.69)	0.001	0.44 (0.28-0.70)	<0.001

*Abbreviations: HBV, hepatitis B virus; HCC, hepatocellular carcinoma; RF, radiofrequency; HR, hazard ratio; CI, confidence interval; INR, international normalized ratio; AFP, alpha-fetoprotein.

Table 5 demonstrates the risk factors for tumor recurrence of HBV-related HCC after RF ablation in sub-analysis based on hepatitis B viral load (HBV DNA levels ≥2,000 IU/mL or <2,000 IU/mL). The use of antiviral treatment after RF ablation was associated with a significantly lower risk of tumor recurrence in patients with a low viral load (HBV DNA <2,000 IU/mL) (HR=0.28 (95% CI: 0.13-0.63), *p*=0.002) as well as in those with a high viral load (HBV DNA ≥2,000 IU/mL) (HR=0.32 (95% CI: 0.21-0.50), *p*<0.001).

**Table 5:** Multivariable analysis of risk factors for tumor recurrence of HBV-related HCC after RF ablation in a subgroup of patients according to hepatitis B viral load

	HBV DNA <2,000 IU/mL		HBV DNA ≥2,000 IU/mL	
	HR (95% CI)	*p*-value	HR (95% CI)	*p*-value
Tumor size (mm)			1.30 (1.01-1.66)	0.043
Prothrombin time (INR)	11.53 (1.53-87.17)	0.018		
AST (U/L)	1.01 (1.00-1.03)	0.034		
Log_10_HBV DNA ( IU/mL)			1.30 (1.06-1.59)	0.013
AFP (≥20 ng/mL)	3.16 (1.63-6.13)	0.001		
Antiviral treatment after RF ablation (+)	0.28 (0.13-0.63)	0.002	0.32 (0.21-0.50)	<0.001

*Abbreviations: HBV, hepatitis B virus; HCC, hepatocellular carcinoma; RF, radiofrequency; HR, hazard ratio; CI, confidence interval; INR, international normalized ratio; AST, aspartate aminotransferase; AFP, alpha-fetoprotein.

### Risk factors for overall survival

Table 6 demonstrates the risk factors for overall survival of patients with HBV-related HCC after RF ablation in the full cohort. Univariable analysis showed that unfavorable overall survival was significantly associated with larger tumor size (HR=1.65 (95% CI: 1.16-2.33), *p*=0.005), lower serum albumin level (HR=0.56 (95% CI: 0.34-0.93), *p*=0.025), higher serum AST level (HR=1.01 (95% CI: 1.00-1.02), *p*=0.039), and Child-Pugh Class B (HR=1.94 (95% CI: 1.04-3.63), *p*=0.038). The use of antiviral treatment after RF ablation had a significantly favorable effect on overall survival (HR=0.48 (95% CI: 0.28-0.84), *p*=0.010). Multivariable analysis revealed that unfavorable overall survival was associated with larger tumor size (HR=1.86 (95% CI: 1.28-2.70), *p*=0.001) and Child-Pugh Class B (HR=2.13 (95% CI: 1.13-4.00), *p*=0.019). The use of antiviral treatment after RF ablation was significantly associated with favorable overall survival (HR=0.44, (95% CI: 0.25-0.77), *p* =0.004).

**Table 6:** Univariable and multivariable analyses of risk factors for overall survival of HBV-related HCC patients after RF ablation in the full cohort (n=228)

	Univariable HR (95% CI)	*p*-value	Multivariable HR (95% CI)	*p*-value
Age (years)	0.99 (0.96-1.02)	0.621		
Men	0.98 (0.68-1.40)	0.439		
Tumor size (mm)	1.65 (1.16-2.33)	0.005	1.86 (1.28-2.70)	0.001
Platelet (x10^3^/mm^3^)	1.00 (0.99-1.00)	0.130		
Prothrombin time (INR)	3.05 (0.65-14.24)	0.156		
Albumin (g/dL)	0.56 (0.34-0.93)	0.025		
Total bilirubin (mg/dL)	1.23 (0.87-1.75)	0.236		
AST (U/L)	1.01 (1.00-1.02)	0.039		
ALT (U/L)	1.00 (1.00-1.01)	0.247		
Log_10_HBV DNA ( IU/mL)	1.03 (0.93-1.14)	0.561		
AFP (≥20 ng/mL)	0.90 (0.53-1.55)	0.706		
HBeAg (+)	1.31 (0.76-2.26)	0.335		
Liver cirrhosis (+)	2.09 (0.75-5.80)	0.158		
Child-Pugh class B	1.94 (1.04-3.63)	0.038	2.13 (1.13-4.00)	0.019
Antiviral treatment after RF ablation (+)	0.48 (0.28-0.84)	0.010	0.44 (0.25-0.77)	0.004

*Abbreviations: HBV, hepatitis B virus; HCC, hepatocellular carcinoma; RF, radiofrequency; HR, hazard ratio; CI, confidence interval; INR, international normalized ratio; AST, aspartate aminotransferase; ALT, alanine aminotransferase; AFP, alpha-fetoprotein; HBeAg, hepatitis B envelope antigen.

## DISCUSSION

Chronic viral hepatitis is the most common cause of HCC [28]. High HBV load is associated with a poor prognosis in patients treated for HBV-related HCC [9, 29]. Multiple lines of evidence indicate that use of oral antiviral treatment improves clinical outcomes in patients with chronic hepatitis B. Liaw *et al.* showed that lamivudine treatment delays disease progression and reduces the incidence of HCC in patients with chronic hepatitis B [30]. Hosaka *et al.* reported that long-term use of entecavir decreases the incidence of HCC in chronic hepatitis B patients [31]. Also, a significant improvement of hepatic fibrosis or cirrhosis was demonstrated in CHB patients who received oral antiviral treatment [32, 33].

Thus, previous studies provided a rationale for suppressing viral replication using antiviral treatment to improve the prognosis of HBV-related HCC patients following curative therapy. The use of oral antiviral treatment was shown to be associated with a lower risk of tumor recurrence and increased overall survival in patients with HBV-related HCC that underwent surgical resection [12, 13]. However, there are a few studies about the effect of oral antiviral treatment on the prognosis of HBV-related HCC patients after RF ablation.

In this study, we investigated long-term clinical outcomes of tumor recurrence and overall survival after RF ablation in HBV-related HCC according to the use of antiviral treatment after ablation. Previous studies reported that the following risk factors are associated with prognosis in HCC patients after RF ablation: age, cirrhosis, Child-Pugh class, tumor size, tumor number, serum tumor marker, and extrahepatic recurrences [15, 16, 34]. Our study showed that several baseline factors were associated with HCC recurrence and overall survival after RF ablation: serum AST, ALT, HBV DNA, AFP, and the presence of HBeAg in full cohort. To minimize the effect of risk factors other than antiviral treatment between the two groups, we analyzed the data using propensity score matching. After matching, there was no significant difference in risk factors between the two groups and analysis using matched cohorts indicated that overall survival is associated with Child-Pugh class, tumor size, and antiviral therapy. The results of our study indicate a clear beneficial effect of oral antiviral treatment on reducing tumor recurrence and improving overall survival of HBV-related HCC after RF ablation.

To our knowledge, there have been two studies exploring the effect of oral antiviral treatment on the prognosis of HBV-related HCC after RF ablation [15, 16]. Those studies showed that antiviral treatment was associated with reduced tumor recurrence in HCC patients after RF ablation. However, there was still insufficient data regarding the effect of HBV suppression by oral antiviral therapy on patients' survival after RF ablation therapy. Recently, Taiwanese investigators reported that nucleos(t)ide analog therapy is associated with a decreased risk of HCC recurrence. However, there was statistically no difference in the 3-year overall mortality between oral antiviral treatment group and no treatment group [16]. Because they used nationwide health insurance research database, detailed information of pretreatment HBV viral load or HBeAg status is missing. HBV factors including HBeAg status and HBV viral load are important known risk factors for clinical outcomes of HBV-related HCC. Our study performed detailed analysis including HBV suppressing effect of oral antiviral treatment in HBV-related HCC patients after RF ablation. We found that oral antiviral treatment not only reduces HCC recurrences but also improves patients' survival after curative RF ablation.

Most clinical guidelines indicate that use of antiviral treatment is recommended in CHB patients with both high HBV DNA level and increased ALT level [35–37]. We analyzed HCC recurrence according to pretreatment HBV DNA levels. Interestingly, the use of antiviral treatment after RF ablation was associated with a significantly lower risk of tumor recurrence in patients with a low viral load as well as in those with a high viral load. Based on our results, we think that the use of oral antiviral treatment is recommended for HBV-related HCC patients harboring any grade of HBV replication after RF ablation. Recently, Sinn *et al*. reported that compensated cirrhotic patients with low viral load (HBV DNA <2000 IU/mL) were not at low risk for HCC, and oral antiviral therapy was associated with lower HCC risk, corroborating our findings [38].

In this study, 19 patients were excluded due to incomplete ablation (3.4%). These patients were considered as a technical RF ablation failure. The causes of technical failure were as follows; difficult to approach tumor location (i.e. hepatic hilum, gallbladder, and dome area) in 14 patients, heat sink effect (because the tumor was adjacent to a large vessel) in 4 patients, and poor cooperation during the RF ablation procedure in 1 patient. For the accurate analysis, we only included the patients with complete ablation and excluded patients who showed a technical RF ablation failure. It is impossible to evaluate whether the antiviral treatment influences HCC recurrence in patients with a technical failure because we cannot regard that those patients received a curative therapy.

Our study has several limitations. First, we conducted the study for the patients with a single HCC with a maximum diameter <5 cm. Although all guidelines recommend RF ablation in HCC with a single nodule (<5 cm) or 2-3 nodules (<3 cm), multiple nodules is an independent factor for tumor recurrence and overall survival [2, 39]. In this study, we aimed to evaluate the prognosis of HBV-related HCC cases after RF ablation according to the use of antiviral treatment rather than tumor characteristics. Therefore, we limited our study to patients with a single nodular HCC. Our results could not be directly translated to patients who undergo RF ablation for multiple HCCs since patients with single tumor were solely included in our study. Second, the starting time point of oral antiviral treatment after RF ablation was not consistent because this study was conducted by a retrospective approach using a historical cohort. Third, this study could not analyze the clinical outcomes after RF ablation according to antiviral agent. The follow-up duration was so different according to antiviral agent because the launch date of each antiviral agent was different in Korea. For example, entecavir was not available in cohort entry date because it was released in 2007 in Korea. Finally, we did not take into account the biological aggressiveness of the tumor because histologic features cannot be obtained in terms of RF ablation therapy, unlike in cases of surgical resection.

In conclusion, the use of oral antiviral treatment was associated with favorable outcomes in terms of tumor recurrence and overall survival in patients with HBV-related HCC after RF ablation therapy.

## PATIENTS AND METHODS

### Study design and patients

**Figure 2:**
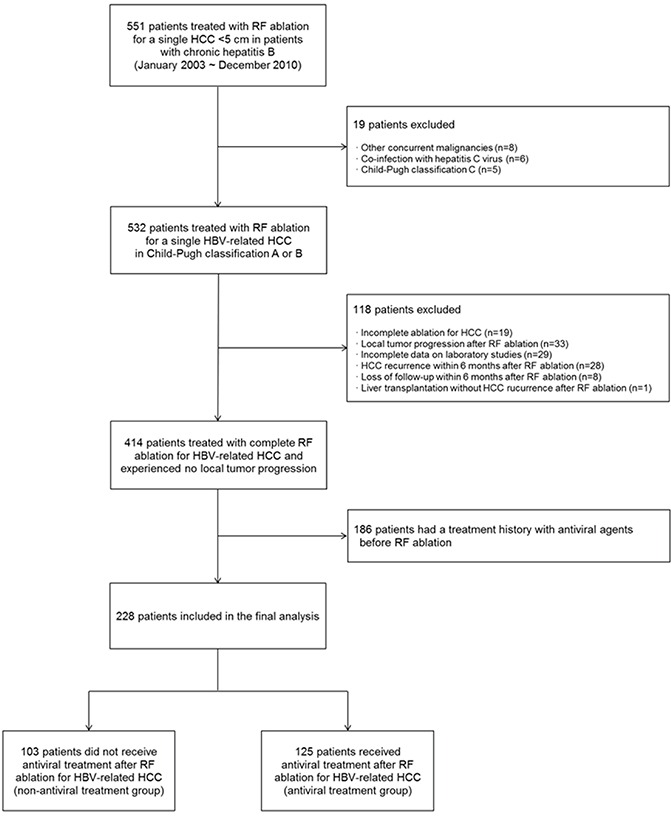
Flow diagram of enrolled patients *Abbreviation: RF, radiofrequency; HCC, hepatocellular carcinoma; HBV, hepatitis B virus.

This is a retrospective study that uses a historical cohort taken from Samsung Medical Center, Seoul, Korea. From January 2003 to December 2010, 551 patients with CHB were treated with RF ablation as a first-line treatment for a single HCC with a maximum diameter of less than 5 cm (Figure 2). Our inclusion criteria for RF ablation in patients with HCC were the same as described in previous studies [15, 17]. Chronic hepatitis B was defined as HBsAg positive for at least 6 months. We excluded the following patients: other concurrent malignancies (n=8); co-infection with hepatitis C virus (n=6); Child-Pugh class C (n=5); incomplete ablation for HCC at computed tomography (CT) obtained immediately after RF ablation (technical failure of RF ablation) (n=19); incomplete data on laboratory studies (n=29); HCC recurrence within 6 months of RF ablation (n=28); lack of follow-up within 6 months of RF ablation (n=8); and liver transplantation without HCC recurrence after RF ablation (n=1). To exclude the potential influence of technical factor for RF ablation procedure on the patient's outcome, the patients with local tumor progression (LTP) during follow up (n=33) were further excluded [18]. In order to evaluate the effect of post-RF ablation antiviral treatment on the prognosis of HBV-related HCC, 186 patients were additionally excluded because of administration of antiviral agent prior to RF ablation. We regarded at least 3 months as a significant duration of antiviral treatment before RF ablation. Finally, we selected 228 HCC patients who were treated using RF ablation for HBV-related HCC. The diagnosis of HCC was done on the basis of American Association for the Study of Liver Diseases (AASLD) guidelines [19]. The indication for the initiation of antiviral treatment was based on Korean Association for the Study of the Liver (KASL) guideline: (i) HBV DNA ≥20,000 IU/mL and serum aminotransferase level (AST or ALT) ≥2 upper limit of normal (ULN) in patients who were HBeAg positive; (ii) HBV DNA ≥2,000 IU/mL and serum aminotransferase level ≥2 ULN in patients who were HBeAg negative; and (iii) HBV DNA ≥2,000 IU/mL and serum aminotransferase level above ULN in cirrhotic patients [20, 21]. This study was approved by the Institutional Review Board of Samsung Medical Center.

### Clinical parameters

We checked the following baseline characteristics at the time of RF ablation therapy: age, sex, tumor size, platelet count, prothrombin time-international normalized ratio (PT-INR), albumin, total bilirubin, aspartate transaminase (AST), alanine transaminase (ALT), alpha-fetoprotein (AFP), HBV DNA level, presence of HBV envelope antigen (HBeAg), presence of liver cirrhosis, Child-Pugh classification, and use of antiviral treatment after RF ablation. Serum HBV DNA level was measured using a hybrid capture assay (Digene Corporation, Gaithersburg, MD, USA) before 2007. After 2007, the COBAS TaqMan hepatitis B virus quantitative test (Roche Molecular Systems Inc., Branchburg, NJ, USA) was used for HBV DNA level testing. Liver cirrhosis was diagnosed histologically (n=32) or clinically according to laboratory and ultrasonographic (US) findings [22]. The antiviral treatment group after RF ablation was defined as those patients who received antiviral treatment for at least 6 months prior to tumor recurrence.

### RF ablation procedure

Before hospitalization for RF ablation, technical feasibility of RF ablation in all patients was assessed on an outpatient basis with planning US [23]. All RF ablation procedures were performed percutaneously under real-time US guidance with local anesthesia and conscious analgesic sedation by M.W.L, H.R. and H.K.L. all of whom had at least 5 years of experience with this procedure prior to the beginning of our study [24]. We used an internally cooled electrode system with generators (Cool-tip RF System, Covidien, Mansfield, MA; VIVA RF ablation System, STARmed, Ilsan, Korea). Either a single or multiple straight electrodes or a cluster electrode were used depending on tumor size and tumor characteristics. We followed the manufacturer's recommended protocol as an algorithm for energy deposition. Strategies for RF ablation concerning optimal electrode type and ablation technique were discussed at an RF ablation team meeting and were based on matching pre-procedural imaging findings with the results of planning US before each treatment. Our therapeutic goal for RF ablation was to achieve an ablative margin of at least 0.5 cm in the normal liver tissue surrounding the tumor, with the exception of subcapsular and perivascular tumors [25]. Procedures were finished when the hyperechoic ablation zone on the US was large enough to cover the entire tumor and the expected ablative margin. For assessment of technical success of the ablation procedure and immediate complications, CT was performed immediately after RF ablation. We followed proposed standardization of terminology and reporting criteria for image-guided tumor ablation throughout the manuscript [26].

### Follow-up after RF ablation

All patients underwent contrast material-enhanced multiphase CT (arterial, portal, and delayed phases) with or without magnetic resonance imaging (MRI) and laboratory tests including tumor marker for assessment of therapeutic outcomes after RF ablation. Tests were performed at 1 month after initial treatment, every 3 months during the first two years after RF ablation, and every 4-6 months thereafter with visits to the outpatient clinic as part of our follow-up protocol. If extrahepatic recurrence (ER) was clinically suspected or unexplained elevation of tumor marker was observed, chest CT, brain MRI, and whole body bone scintigraphy with or without positron emission tomography-CT were performed. If recurrent tumor was identified during the follow-up period, patients were treated with RF ablation, transarterial chemoembolization, sorafenib, radiation therapy, or conservative treatment, based on the results of a multidisciplinary discussion for HCC treatment that depended on the characteristics of the tumor recurrence, liver function, and the general condition of the patient. Tumor recurrence after initial RF ablation was classified into three subtypes according to the reporting guidelines [26]: LTP, intrahepatic distant recurrence (IDR), and ER. In our study, IDR and ER were defined as tumor recurrence, whereas LTP was excluded because our aim was to investigate the role of antiviral treatment on HCC recurrence after RF ablation.

### Statistical analysis

Categorical variables are described as frequencies and percentages. Continuous variables are presented as mean ± standard deviation (SD) and median with interquartile range for parametric and non-parametric variables, respectively. The probabilities of tumor recurrence and overall survival were assessed according to the use of antiviral treatment. The full cohort consisted of 125 patients who received antiviral treatment and 103 patients who did not. We used a 1-to-n propensity score matching from a logistic regression to minimize the difference in baseline covariates between two groups. We performed propensity score matching in consideration of all variables in Table 1 (age, gender, tumor size, platelet count, prothrombin time, albumin, total bilirubin, AST, ALT, Log_10_HBV DNA, AFP, HBeAg status, LC, and Child-Pugh class). Propensity score matching was done using the caliper matching, and the caliper width was 0.2 of the standard deviation of the logit of the propensity score. To assess the balance of variables used matching, standardized mean difference was checked. A value <0.10 indicates a very small difference, 0.1–0.3 indicates a small difference, 0.3–0.5 indicates a moderate difference, and >0.5 indicates a large difference [27]. The matched cohort consisted of 103 patients from the antiviral treatment group and 62 from the non-antiviral treatment group. Kaplan-Meier curves of the two patient groups were used to estimate time to recurrence and overall survival, and were compared using a log-rank test and a weighted log-rank test for the full cohort and the matched cohort, respectively. The follow-up duration for survival analysis was defined as the interval between the first RF ablation and either the incidence of event or the last visit to the outpatient clinic before August 31, 2014. Univariable and multivariable Cox proportional hazards models were used to assess the risk factors for tumor recurrence and overall survival after RF ablation in the full cohort. The variables used for multivariable analysis were selected on the basis of statistical significance in the univariable analysis (*p*<0.20) or clinical significance. Multivariable analysis was performed using a forward conditional stepwise procedure to avoid multicollinearity. A *p*-value less than 0.05 in a 2-sided test is regarded as statistically significant. These analyses were conducted using SAS version 9.4 (SAS Institute, Cary, NC) and R 2.10.0 (Vienna, Austria; http://www.R-project.org).

### What is current knowledge

Recent studies show that the use of oral antiviral treatment improves clinical outcomes in patients with chronic hepatitis B. In particular, oral antiviral treatment has a favorable outcome on the prevention of hepatitis B virus (HBV)-related hepatocellular carcinoma (HCC). Oral antiviral treatment results in favorable outcomes in terms of tumor recurrence and overall survival of HBV-related HCC after surgical resection.

### What is new here

Oral antiviral treatment reduces tumor recurrence and improves overall survival in patients with HBV-related HCC after radiofrequency (RF) ablation. Virological suppression by antiviral treatment is important for the prognosis of HCC patients with chronic hepatitis B after RF ablation.

## SUPPLEMENTARY TABLES AND FIGURE





## Abbreviations

HCC: hepatocellular carcinoma
RF: radiofrequency
HBV: hepatitis B virus
CHB: chronic hepatitis B
HBsAg: hepatitis B virus surface antigen
HBeAg: hepatitis B virus envelope antigen
nucleos(t)ide: nucleoside/nucleotide
CT: computed tomography
PT-INR: prothrombin time-international normalized ratio
AST: aspartate transaminase
ALT: alanine transaminase
AFP: alpha-fetoprotein
US: ultrasonography
MRI: magnetic resonance imaging
LTP: local tumor progression
IDR: intrahepatic distant recurrence
ER: extrahepatic recurrence
SD: standard deviation
CI: confidence interval

## References

[1] Jemal A, Bray F, Center MM, Ferlay J, Ward E, Forman D (2011). Global cancer statistics. CA Cancer J Clin.

[2] European Association For The Study Of The L, European Organisation For R and Treatment Of C (2012). EASL-EORTC clinical practice guidelines: management of hepatocellular carcinoma. Journal of hepatology.

[3] Forner A, Llovet JM, Bruix J (2012). Hepatocellular carcinoma. Lancet.

[4] Komorizono Y, Oketani M, Sako K, Yamasaki N, Shibatou T, Maeda M, Kohara K, Shigenobu S, Ishibashi K, Arima T (2003). Risk factors for local recurrence of small hepatocellular carcinoma tumors after a single session, single application of percutaneous radiofrequency ablation. Cancer.

[5] N'Kontchou G, Mahamoudi A, Aout M, Ganne-Carrie N, Grando V, Coderc E, Vicaut E, Trinchet JC, Sellier N, Beaugrand M, Seror O (2009). Radiofrequency ablation of hepatocellular carcinoma: long-term results and prognostic factors in 235 Western patients with cirrhosis. Hepatology.

[6] Yang B, Zou J, Xia J, Ren Z, Gan Y, Wang Y, Zhang B, Ge N, Wang D, Chen Y, Chen R, Li L, Ye S, Wang X (2011). Risk factors for recurrence of small hepatocellular carcinoma after long-term follow-up of percutaneous radiofrequency ablation. European journal of radiology.

[7] Facciorusso A, Del Prete V, Antonino M, Neve V, Crucinio N, Di Leo A, Carr BI, Barone M (2014). Serum ferritin as a new prognostic factor in hepatocellular carcinoma patients treated with radiofrequency ablation. Journal of gastroenterology and hepatology.

[8] Hung IF, Poon RT, Lai CL, Fung J, Fan ST, Yuen MF (2008). Recurrence of hepatitis B-related hepatocellular carcinoma is associated with high viral load at the time of resection. The American journal of gastroenterology.

[9] Qu LS, Jin F, Huang XW, Shen XZ (2010). High hepatitis B viral load predicts recurrence of small hepatocellular carcinoma after curative resection. Journal of gastrointestinal surgery.

[10] Sohn W, Paik YH, Kim JM, Kwon CH, Joh JW, Cho JY, Gwak GY, Choi MS, Lee JH, Koh KC, Paik SW, Yoo BC (2014). HBV DNA and HBsAg levels as risk predictors of early and late recurrence after curative resection of HBV-related hepatocellular carcinoma. Annals of surgical oncology.

[11] Sohn W, Paik YH, Lee MW, Rhim H, Lim HK, Cho JY, Gwak GY, Choi MS, Lee JH, Koh KC, Paik SW, Yoo BC (2014). Predisposing factors for recurrence of HBV-related small hepatocellular carcinoma after percutaneous radiofrequency ablation. Scandinavian journal of gastroenterology.

[12] Wu CY, Chen YJ, Ho HJ, Hsu YC, Kuo KN, Wu MS, Lin JT (2012). Association between nucleoside analogues and risk of hepatitis B virus-related hepatocellular carcinoma recurrence following liver resection. Jama.

[13] Yin J, Li N, Han Y, Xue J, Deng Y, Shi J, Guo W, Zhang H, Wang H, Cheng S, Cao G (2013). Effect of antiviral treatment with nucleotide/nucleoside analogs on postoperative prognosis of hepatitis B virus-related hepatocellular carcinoma: a two-stage longitudinal clinical study. Journal of clinical oncology.

[14] Huang G, Lau WY, Wang ZG, Pan ZY, Yuan SX, Shen F, Zhou WP, Wu MC (2015). Antiviral therapy improves postoperative survival in patients with hepatocellular carcinoma: a randomized controlled trial. Annals of surgery.

[15] Kim YS, Lim HK, Rhim H, Lee MW, Choi D, Lee WJ, Paik SW, Koh KC, Lee JH, Choi MS, Gwak GY, Yoo BC (2013). Ten-year outcomes of percutaneous radiofrequency ablation as first-line therapy of early hepatocellular carcinoma: analysis of prognostic factors. Journal of hepatology.

[16] Lee TY, Lin JT, Zeng YS, Chen YJ, Wu MS, Wu CY (2016). Association between nucleos(t)ide analog and tumor recurrence in hepatitis B virus-related hepatocellular carcinoma after radiofrequency ablation. Hepatology.

[17] Kang TW, Lee MW, Hye MJ, Song KD, Lim S, Rhim H, Lim HK, Cha DI (2014). Percutaneous radiofrequency ablation of hepatic tumours: Factors affecting technical failure of artificial ascites formation using an angiosheath. Clinical radiology.

[18] Kim YS, Lee WJ, Rhim H, Lim HK, Choi D, Lee JY (2010). The minimal ablative margin of radiofrequency ablation of hepatocellular carcinoma (> 2 and < 5 cm) needed to prevent local tumor progression: 3D quantitative assessment using CT image fusion. AJR American journal of roentgenology.

[19] Bruix J, Sherman M (2005). Management of hepatocellular carcinoma. Hepatology.

[20] Korean Association for the Study of the L (2012). KASL Clinical Practice Guidelines: Management of chronic hepatitis B. Clinical and molecular hepatology.

[21] Korean Association for the Study of the L (2016). KASL clinical practice guidelines: management of chronic hepatitis B. Clinical and molecular hepatology.

[22] Berzigotti A, Castera L (2013). Update on ultrasound imaging of liver fibrosis. Journal of hepatology.

[23] Rhim H, Choi D, Kim YS, Lim HK, Choe BK (2010). Ultrasonography-guided percutaneous radiofrequency ablation of hepatocellular carcinomas: a feasibility scoring system for planning sonography. European journal of radiology.

[24] Kang TW, Lim HK, Lee MW, Kim YS, Choi D, Rhim H (2014). Perivascular versus nonperivascular small HCC treated with percutaneous RF ablation: retrospective comparison of long-term therapeutic outcomes. Radiology.

[25] Kang TW, Kim JM, Rhim H, Lee MW, Kim YS, Lim HK, Choi D, Song KD, Kwon CH, Joh JW, Paik SW, Paik YH, Ahn JH (2015). Small Hepatocellular Carcinoma: Radiofrequency Ablation versus Nonanatomic Resection--Propensity Score Analyses of Long-term Outcomes. Radiology.

[26] Ahmed M, Solbiati L, Brace CL, Breen DJ, Callstrom MR, Charboneau JW, Chen MH, Choi BI, de Baere T, Dodd GD, Dupuy DE, Gervais DA, Gianfelice D, Gillams AR, Lee FT, Leen E (2014). Image-guided tumor ablation: standardization of terminology and reporting criteria--a 10-year update. Radiology.

[27] Burnand B, Kernan WN, Feinstein AR (1990). Indexes and boundaries for “quantitative significance” in statistical decisions. Journal of clinical epidemiology.

[28] El-Serag HB (2012). Epidemiology of viral hepatitis and hepatocellular carcinoma. Gastroenterology.

[29] Yang T, Lu JH, Zhai J, Lin C, Yang GS, Zhao RH, Shen F, Wu MC (2012). High viral load is associated with poor overall and recurrence-free survival of hepatitis B virus-related hepatocellular carcinoma after curative resection: a prospective cohort study. European journal of surgical oncology.

[30] Liaw YF, Sung JJ, Chow WC, Farrell G, Lee CZ, Yuen H, Tanwandee T, Tao QM, Shue K, Keene ON, Dixon JS, Gray DF, Sabbat J, Cirrhosis Asian Lamivudine Multicentre Study G (2004). Lamivudine for patients with chronic hepatitis B and advanced liver disease. The New England journal of medicine.

[31] Hosaka T, Suzuki F, Kobayashi M, Seko Y, Kawamura Y, Sezaki H, Akuta N, Suzuki Y, Saitoh S, Arase Y, Ikeda K, Kobayashi M, Kumada H (2013). Long-term entecavir treatment reduces hepatocellular carcinoma incidence in patients with hepatitis B virus infection. Hepatology.

[32] Chang TT, Liaw YF, Wu SS, Schiff E, Han KH, Lai CL, Safadi R, Lee SS, Halota W, Goodman Z, Chi YC, Zhang H, Hindes R, Iloeje U, Beebe S, Kreter B (2010). Long-term entecavir therapy results in the reversal of fibrosis/cirrhosis and continued histological improvement in patients with chronic hepatitis B. Hepatology.

[33] Marcellin P, Gane E, Buti M, Afdhal N, Sievert W, Jacobson IM, Washington MK, Germanidis G, Flaherty JF, Schall RA, Bornstein JD, Kitrinos KM, Subramanian GM, McHutchison JG, Heathcote EJ (2013). Regression of cirrhosis during treatment with tenofovir disoproxil fumarate for chronic hepatitis B: a 5-year open-label follow-up study. Lancet.

[34] Shiina S, Tateishi R, Arano T, Uchino K, Enooku K, Nakagawa H, Asaoka Y, Sato T, Masuzaki R, Kondo Y, Goto T, Yoshida H, Omata M, Koike K (2012). Radiofrequency ablation for hepatocellular carcinoma: 10-year outcome and prognostic factors. The American journal of gastroenterology.

[35] Lok AS, McMahon BJ (2007). Chronic hepatitis B. Hepatology.

[36] Liaw YF, Leung N, Kao JH, Piratvisuth T, Gane E, Han KH, Guan R, Lau GK, Locarnini S, Chronic Hepatitis BGWPotA-PAftSotL (2008). Asian-Pacific consensus statement on the management of chronic hepatitis B: a 2008 update. Hepatology international.

[37] European Association For The Study Of The L (2012). EASL clinical practice guidelines: Management of chronic hepatitis B virus infection. Journal of hepatology.

[38] Sinn DH, Lee J, Goo J, Kim K, Gwak GY, Paik YH, Choi MS, Lee JH, Koh KC, Yoo BC, Paik SW (2015). Hepatocellular carcinoma risk in chronic hepatitis B virus-infected compensated cirrhosis patients with low viral load. Hepatology.

[39] Bruix J, Sherman M, American Association for the Study of Liver D (2011). Management of hepatocellular carcinoma: an update. Hepatology.

