# Supplementary material for: Effect of oral antiviral treatment on long-term outcomes of radiofrequency ablation therapy for hepatitis B virus-related hepatocellular carcinoma
Source: Oncotarget. 2016 Jun 14;7(30):47794–807. doi: 10.18632/oncotarget.10026 (PMC5216979; doi:10.18632/oncotarget.10026)
Supplement: Supplementary file 1 [file oncotarget-07-47794-s001.pdf]

Effect of oral antiviral treatment on long-term outcomes of radiofrequency ablation therapy for hepatitis B virus-related hepatocellular carcinoma

SUPPLEMENTARY TABLES AND FIGURE

Supplementary Table S1: Baseline characteristics of the patients treated with antiviral treatment (n=125).

	ETV (n=68)	LAM (n=45)	CLV (n=7)	ADV (n=4)	TDF (n=1)
Age (years)	57.0 ± 8.6	53.4 ± 9.0	50.3 ± 9.3	46.5 ± 6.9	60.0
Gender					
women	18 (27%)	15 (33%)	1 (14%)	1 (25%)	0 (0%)
men	50 (73%)	30 (67%)	6 (86%)	3 (75%)	1 (100%)
Tumor size (mm)	2.1 ± 0.7	2.4 ± 0.8	2.1 ± 0.8	2.0 ± 0.4	1.6
Platelet ($x10^3/mm^3$)	108.9 ± 37.4	111.6 ± 55.4	130.1 ± 56.5	71.8 ± 12.4	152.0
Prothrombin time (INR)	1.22 ± 0.14	1.23 ± 0.18	1.15 ± 0.06	1.44 ± 0.26	1.11
Albumin (g/dL)	3.6 ± 0.5	3.5 ± 0.6	3.8 ± 0.3	3.1 ± 0.6	3.6
Total bilirubin (mg/dL)	0.8 ± 0.4	1.1 ± 0.7	0.7 ± 0.5	2.0 ± 1.5	0.6
AST (U/L)	47.0 ± 20.1	63.5 ± 38.6	41.1 ± 10.4	101.5 ± 69.5	32.0
ALT (U/L)	44.1 ± 23.9	63.1 ± 58.1	39.9 ± 19.8	90.3 ± 77.3	30.0
Log_{10}HBV DNA (IU/mL)	5.4 ± 1.8	5.3 ± 2.2	5.6 ± 1.0	4.9 ± 2.2	0
AFP (ng/mL)					
<20	26 (38%)	17 (38%)	3 (43%)	0 (0%)	1 (100%)
≥20	42 (62%)	28 (62%)	4 (57%)	4 (100%)	0 (0%)
HBeAg (N, %)					
negative	37 (54%)	24 (53%)	1 (14%)	1 (25%)	1 (100%)
positive	31 (46%)	21 (47%)	6 (86%)	3 (75%)	0 (0%)
Liver cirrhosis (N, %)					
absence	15 (22%)	4 (9%)	2 (29%)	0 (0%)	0 (0%)
presence	53 (78%)	41 (91%)	5 (71%)	4 (100%)	1 (100%)
Child-Pugh class (N, %)					
A	61 (90%)	36 (80%)	7 (100%)	2 (50%)	1 (100%)
B	7 (10%)	9 (20%)	0 (0%)	2 (50%)	0 (0%)

Data are presented as the mean ± standard deviation or number of patients (percentages in parentheses).
*Abbreviations: ETV, entecavir; LAM, lamivudine; CLV, clevudine; ADV, adefovir; TDF, tenofovir; INR, international normalized ratio; AST, aspartate aminotransferase; ALT, alanine aminotransferase; HBV, hepatitis B virus; AFP, alpha-fetoprotein; HBeAg, hepatitis B envelope antigen.

Supplementary Table S2: Recurrence events and therapeutic modalities at the end of the follow-up

See Supplementary File: 1

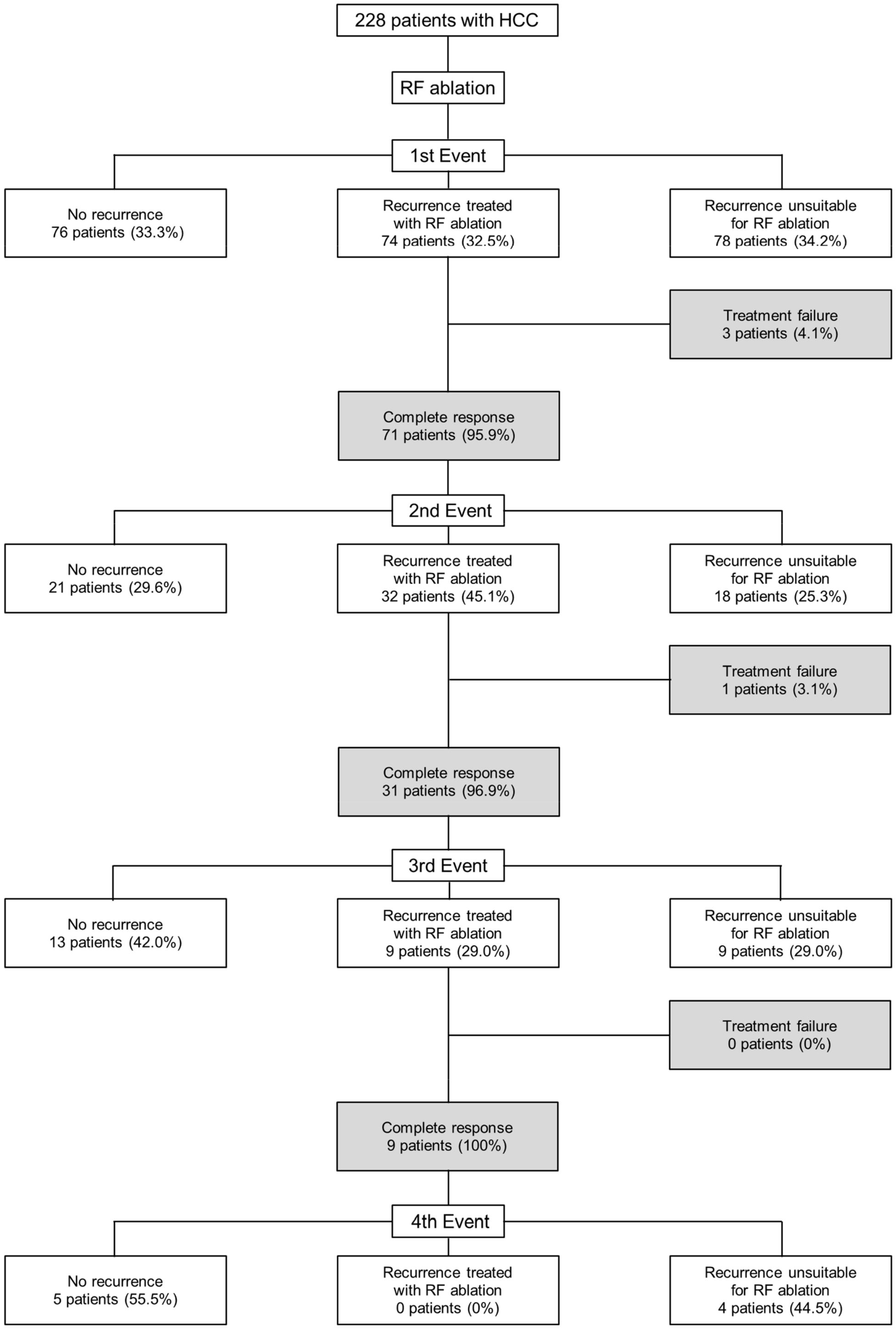

Supplementary Figure S1: Summary of follow-up events. Recurrent HCC were treated with RF ablation when the patient met the criteria of the initial therapy. Unless RF ablation was done, the patients were treated by other therapeutic modalities.
Abbreviations: HCC, hepatocellular carcinoma; RF, radiofrequency.
