# Supplementary material for: Effect of oral antiviral treatment on long-term outcomes of radiofrequency ablation therapy for hepatitis B virus-related hepatocellular carcinoma
Source: Oncotarget. 2016 Jun 14;7(30):47794–807. doi: 10.18632/oncotarget.10026 (PMC5216979; doi:10.18632/oncotarget.10026)
Supplement: Supplementary file 2 [file oncotarget-07-47794-s002.docx]

Supplement table 2. Recurrence events and therapeutic modalities at the end of the follow-up

|  |  |  |  | Treatment No. (%) | | | | | | | | | |
| --- | --- | --- | --- | --- | --- | --- | --- | --- | --- | --- | --- | --- | --- |
|  |  | No. (%) | RF ablation | | TACE | Surgical resection | LT | Sorafenib | Radiation therapy | Metassectomy | BSC | *None | Lost to follow-up |
| 1^st^ event |  |  |  | |  |  |  |  |  |  |  |  |  |
|  | No event | 76/228 (33.3%) |  | |  |  |  |  |  |  |  |  |  |
|  | Recurrence | 152/228 (66.7%) |  | |  |  |  |  |  |  |  |  |  |
|  | Local tumor progression | 0 |  | |  |  |  |  |  |  |  |  |  |
|  | Intrahepatic distant recurrence | 146 | 74  (50.7%) | | 64 (43.8%) | 3  (2.1%) | 1  (0.6%) | 0 | 0 | 0 | 0 | 2  (1.4%) | 2  (1.4%) |
|  | Extrahepatic metastasis | 6 | 0 | | 0 | 0 | 0 | 2  (33.3%) | 2  (33.3%) | 1  (16.6%) | 1  (16.7%) | 0 | 0 |
|  | Total treated |  | 74 | |  |  |  |  |  |  |  |  |  |
|  | Complete response |  | 71 | |  |  |  |  |  |  |  |  |  |
|  | Treatment failure |  | 3 | |  |  |  |  |  |  |  |  |  |
| 2^nd^ event |  |  |  | |  |  |  |  |  |  |  |  |  |
|  | No event | 21/71 (29.6%) |  | |  |  |  |  |  |  |  |  |  |
|  | Recurrence | 50/71 (71.4%) |  | |  |  |  |  |  |  |  |  |  |
|  | Local tumor progression | 5 | 4  (80.0%) | | 1  (20.0%) | 0 | 0 | 0 | 0 | 0 | 0 | 0 | 0 |
|  | Intrahepatic distant recurrence | 45 | 28  (62.2%) | | 17  (37.8%) | 0 | 0 | 0 | 0 | 0 | 0 | 0 | 0 |
|  | Extrahepatic metastasis | 0 |  | |  |  |  |  |  |  |  |  |  |
|  | Total treated |  | 32 | |  |  |  |  |  |  |  |  |  |
|  | Complete response |  | 31 | |  |  |  |  |  |  |  |  |  |
|  | Treatment failure |  | 1 | |  |  |  |  |  |  |  |  |  |
| 3^rd^ event |  |  |  | |  |  |  |  |  |  |  |  |  |
|  | No event | 13/31 (41.9%) |  | |  |  |  |  |  |  |  |  |  |
|  | Recurrence | 18/31 (58.1%) |  | |  |  |  |  |  |  |  |  |  |
|  | Local tumor progression | 2 | 2  (100%) | | 0 | 0 | 0 | 0 | 0 | 0 | 0 | 0 | 0 |
|  | Intrahepatic distant recurrence | 14 | 7  (50.0%) | | 7  (50.0%) | 0 | 0 | 0 | 0 | 0 | 0 | 0 | 0 |
|  | Extrahepatic metastasis | 2 | 0 | | 0 | 0 | 0 | 1  (50.0%) | 1  (50.0%) | 0 | 0 | 0 | 0 |
|  | Total treated |  | 9 | |  |  |  |  |  |  |  |  |  |
|  | Complete response |  | 9 | |  |  |  |  |  |  |  |  |  |
|  | Treatment failure |  | 0 | |  |  |  |  |  |  |  |  |  |
| 4^th^ event |  |  |  | |  |  |  |  |  |  |  |  |  |
|  | No event | 5/9 (55.5%) |  | |  |  |  |  |  |  |  |  |  |
|  | Recurrence | 4/9 (44.5%) |  | |  |  |  |  |  |  |  |  |  |
|  | Local tumor progression | 0 |  | |  |  |  |  |  |  |  |  |  |
|  | Intrahepatic distant recurrence | 4 | 0 | | 4  (100%) | 0 | 0 | 0 | 0 | 0 | 0 | 0 | 0 |
|  | Extrahepatic metastasis | 0 |  | |  |  |  |  |  |  |  |  |  |
|  | Total treated |  |  | |  |  |  |  |  |  |  |  |  |
|  | Complete response |  |  | |  |  |  |  |  |  |  |  |  |
|  | Treatment failure |  |  | |  |  |  |  |  |  |  |  |  |

*’None’ is that the patients didn’t want any therapy for recurrent HCC.

Abbreviations: RF, radiofrequency; TACE, transarterial chemoembolization; LT, liver transplantation; BSC, best supportive care.
